# Benznidazole Treatment: Time- and Dose-Dependence Varies with the *Trypanosoma cruzi* Strain

**DOI:** 10.3390/pathogens10060729

**Published:** 2021-06-09

**Authors:** Kátia da Silva Fonseca, Luísa Perin, Nívia Carolina Nogueira de Paiva, Beatriz Cristiane da Silva, Thays Helena Chaves Duarte, Flávia de Souza Marques, Guilherme de Paula Costa, Israel Molina, Rodrigo Correa-Oliveira, Paula Melo de Abreu Vieira, Cláudia Martins Carneiro

**Affiliations:** 1 Laboratory of Immunopathology, Nucleus of Biological Sciences Research, Federal University of Ouro Preto, Ouro Preto 35400-000, Brazil; katia.fonseca@gmail.com (K.d.S.F.); perindemelo@gmail.com (L.P.); niviacarolinanp@ufop.edu.br (N.C.N.d.P.); luisaperin@yahoo.com (B.C.d.S.); guilherme.costa@aluno.ufop.edu.br (G.d.P.C.); israelmolina@ymail.com (I.M.); correa@cpqrr.fiocruz.br (R.C.-O.); paula@ufop.edu.br (P.M.d.A.V.); 2 Laboratory of Morphopathology, Department of Biological Sciences, Nucleus of Biological Sciences Research, Institute of Exact and Biological Sciences, Federal University of Ouro Preto, Ouro Preto 35400-000, Brazil; thays.duarte@aluno.ufop.edu.br (T.H.C.D.); flavia.marques1@aluno.ufop.edu.br (F.d.S.M.); 3 Tropical Medicine and International Health Unit, Department of Infectious Diseases, Vall d’Hebron University Hospital, Universitat Autònoma de Barcelona, PROSICS Barcelona, 08035 Barcelona, Spain; 4 Laboratory of Cellular and Molecular Immunology, René Rachou Research Center, Oswaldo Cruz Foundation, Belo Horizonte 30190-002, Brazil; 5 Department of Clinical Analysis, School of Pharmacy, Federal University of Ouro Preto, Ouro Preto 35400-000, Brazil

**Keywords:** chagas disease, *Trypanosoma cruzi*, benznidazole, therapeutic strategies, mice

## Abstract

As the development of new drugs for Chagas disease is not a priority due to its neglected disease status, an option for increasing treatment adherence is to explore alternative treatment regimens, which may decrease the incidence of side effects. Therefore, we evaluated the efficacy of different therapeutic schemes with benznidazole (BNZ) on the acute and chronic phases of the disease, using mice infected with strains that have different BNZ susceptibilities. Our results show that the groups of animals infected by VL-10 strain, when treated in the chronic phase with a lower dose of BNZ for a longer period of time (40 mg/kg/day for 40 days) presented better treatment efficacy than with the standard protocol (100 mg/kg/day for 20 days) although the best result in the treatment of the animals infected by the VL-10 strain was with100 mg/kg/day for 40 days. In the acute infection by the Y and VL-10 strains of *T. cruzi*, the treatment with a standard dose, but with a longer time of treatment (100 mg/kg/day for 40 days) presented the best results. Given these data, our results indicate that for BNZ, the theory of dose and time proportionality does not apply to the phases of infection.

## 1. Introduction

Chagas disease is a neglected protozoan disease that affects more than 6 million people worldwide and is endemic in Latin America [1]. It is caused by the hemoflagellate parasite *Trypanosoma cruzi* (*T. cruzi*)*,* belonging to the order Kinetoplastida and family Tripanosomatidae, whose main invertebrate hosts and vectors of Chagas disease, are hematophagous insects of the subfamily Triatominae [2,3]. *T. cruzi* has a high genetic heterogeneity and using different molecular markers, genotyping of the strains of the parasite was carried out, in order to classify it in several subgroups (or discrete typing units, DTUs) termed TcI–TcVI. However, studies have suggested that the DTUs would have the status of subspecies [3].

Benznidazole (BNZ), the reference drug in the treatment of Chagas disease, shows satisfactory results in the acute and recent chronic phases of infection, but causes numerous side effects that lead to treatment interruption by significant numbers of patients [4,5,6,7,8,9,10]. There are few efforts to discover new drugs for Chagas disease, therefore, an option for increasing treatment adherence is to explore alternative treatment regimens with BNZ, which could decrease the incidence of side effects.

A number of studies have suggested that it is possible to reduce the dose of BNZ currently used for treatment of Chagas disease. First, researchers reported that 20% of adults who prematurely abandoned BNZ treatment due to adverse effects, displayed negative serology for *T. cruzi* [11]. Furthermore, although plasma concentrations of BNZ in children subjected to the treatment are lower than in adults, the treatment efficacy is proven, suggesting that the BNZ dose used in the treatment of adults could be decreased and still generate similar exposure to that observed in children, maintaining efficacy and reducing side effects [12]. A prospective study of population pharmacokinetics showed, through dose simulations, that it is possible to decrease the dose of BNZ in relation to the conventional dose while keeping the plasma concentration within the recommended target range, indicating the need to evaluate whether lower doses could be as efficient as the conventional dose [13]. Moreover, animals subjected to intermittent treatment that reduces the total dose of BNZ can be cured of *T. cruzi* infection [14]. However, a recent study showed that to obtain parasitological cure in mice chronically infected with resistant *T. cruzi* strains it is necessary to increase the dose of BNZ and extend the length of treatment [15]. Lastly, mice in the acute phase of *T. cruzi* infection, when treated with BNZ, have a dependent dose-time response profile [16].

It still remains necessary to evaluate other therapeutic regimens by altering dose and time of treatment simultaneously and, mainly, to perform the evaluation in the chronic period of the infection, the phase when most patients are treated. Therefore, the objective of this work was to evaluate the influence of the dose and time treatment in curing mice in the acute and chronic phases of the infection against strains with different susceptibilities to BNZ treatment.

## 2. Materials and Methods

### 2.1. Animals and Ethics

One hundred and twenty Swiss mice, 30 days old, weighing 18–25 g, and provided by the Animal Science Center of the Federal University of Ouro Preto (CCA, UFOP), were kept under control conditions with regular cycles of light, temperature of 23 ± 2 °C, and availability of food and water ad libitum. All procedures were performed according to ethical principles recommended by the National Council for Control of Animal Experimentation (CONCEA) and approved by the ethics committee of Federal University of Ouro Preto (Protocol CEUA-UFOP number: 2016/33). 

The mice were monitored daily by the researchers, who have extensive experience, so that visible physical changes were communicated to the veterinarian responsible for the sector, in order to determine the appropriate handling of the animal. We emphasize that some physical changes are characteristic of infection by *Trypanosoma cruzi*, such as weight loss and bristly fur, and in none of the evaluations carried out by the veterinarian throughout the project, were the animals euthanized.

### 2.2. Therapeutic Schemes

Mice were inoculated with 5 × 10^3^ of the blood trypomastigote forms of *T. cruzi*, all trypomastigotes were obtained from the blood samples of infected albino Swiss mice at the peak of parasitemia. These animals were divided into three groups: infected with Y strain (DTU II) and treated in the acute phase (n = 40); infected with VL-10 strain (DTU II) and treated in the acute phase (n = 40); and infected with VL-10 strain and treated in the chronic phase (n = 40). To exclude variables, the inoculum was the same for all phases/strains. Y strain was not observed in chronic phase because the animals are not able to survive until chronic phase with this inoculum.

In the acute phase and in the chronic phase of this experiment, groups of eight animals were analyzed: (1) infected and untreated; (2) infected and treated with BNZ 100 mg/kg/day for 20 days; (3) infected and treated with BNZ 100 mg/kg/day for 40 days; (4) infected and treated with BNZ 40 mg/kg/day for 20 days; and (5) infected and treated with BNZ 40 mg/kg/day for 40 days. BNZ was prepared by pulverization of 100 mg tablets (Pharmaceutical Laboratory of Pernambuco State, Recife, Brazil), suspended in an aqueous solution of methylcellulose 0.5% (Sigma-Aldrich, St. Louis, MO, USA), and each animal received, according to body weight, the suspension of the drug by gavage. The treatment was initiated in the acute phase after confirmation of infection. The mice in the untreated group received oral gavage with water and the treated groups received BNZ suspended in a solution of water and methylcellulose. As this drug has low solubility in water [17], this strategy is necessary to enable greater homogeneity of the suspended substance, being the methylcellulose the suspending agent most used in pharmaceutical preparations, including in studies carried out by Chagas disease research groups [16,18,19]. 

Animals were evaluated in relation to survival, fresh blood examination (FBE), parasitemia, real-time polymerase chain reaction (qPCR) of blood five days after immunosuppression with cyclophosphamide (CyI), necropsy and qPCR of heart and colon 30 days after CyI. Treatment by gavage of animals in the chronic phase was initiated 120 days after infection. The same parameters evaluated in the acute phase were used in the chronic phase. The experiments lasted from 78 days (acute phase—20 days) to 213 days (chronic phase—40 days).

### 2.3. Cure Criteria

For therapeutic evaluation, the analysis included: survival, parasitemia, and cure through FBE and qPCR after CyI. Only animals that were negative in both tests were considered cured [20].

#### 2.3.1. Survival

Animals were followed daily after inoculum up to 30 days after the end of the treatment and mortality was recorded [20].

#### 2.3.2. Parasitemia

Parasitemia was evaluated daily from the 4th day after infection until examination was negative for five consecutive days, following the adapted methodology [21]. 

#### 2.3.3. Immunosuppression and Fresh Blood Examination

Thirty days after the end of treatment all mice were subjected to CyI. Parasitemia was evaluated daily during this process and until five days after CyI, adapted methodology [22]. 

#### 2.3.4. Real-Time Polymerase Chain Reaction (qPCR)

##### qPCR in Total Blood

Peripheral blood of mice was collected five days after the end of CyI, stored in Eppendorf tubes containing EDTA (anticoagulant), and kept in ice until extraction. Samples were subjected to procedures of DNA extraction using DNeasy^®^ Blood and Tissue Kit (Qiagen^®^, Hilden, Germany), following the manufacturer’s recommendations.

##### qPCR in Tissue

Fragments of heart and colon were collected during necropsy and then subjected to procedures of DNA extraction using WizardTM Genomic DNA Purification Kit (Promega, Madison, WI, USA), following the manufacturer’s recommendations. 

### 2.4. Statistical Analyses

Statistical analyses of the data were performed using GraphPad Prisma software (GraphPad Software, San Diego, CA, USA). The parasitemia was expressed as mean ± standard error. To compare the area under the curve (AUC), Kruskal-Wallis test/Dunn’s multiple comparisons test was used, and Kruskal-Wallis test/Dunn’s multiple comparisons test were used to compare maximum parasitemia peak.

The data were evaluated by contingency table, Fisher exact test, and odds ratio to compare the percentage of cure among the different groups. Differences in mean values were considered significant at *p* < 0.05.

## 3. Results

**Figure 1:**
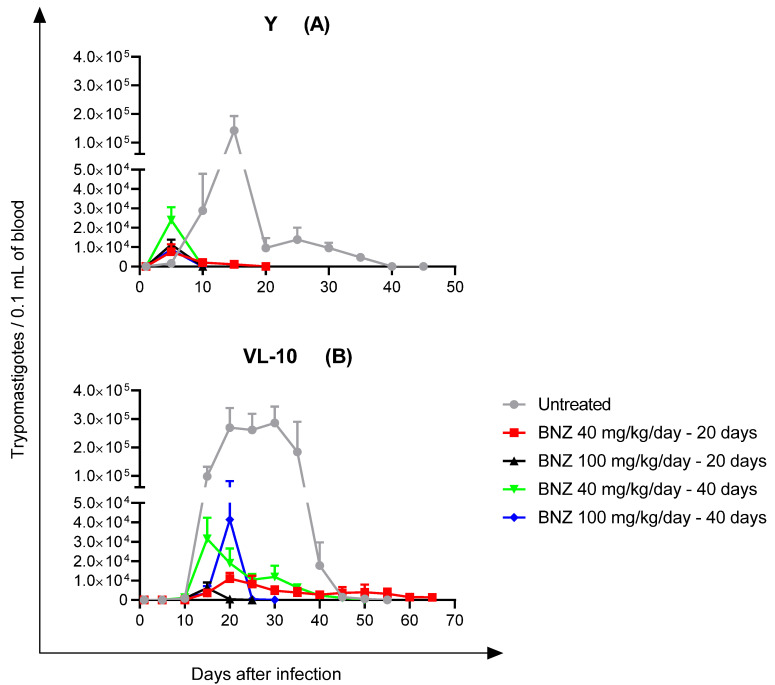
Effect of different benznidazole (BNZ) therapeutic protocols on parasitemia by *Trypanosoma cruzi* strains with different susceptibilities to BNZ. (**A**) Y: partially susceptible, and (**B**) VL-10: resistant. Standard treatment for mice: BNZ 100 mg/kg/day for 20 days. Parasitemia was measured by an adapted version of the Brener method.

Mice infected with Y strain and treated with all protocols showed a reduction in the parasitemia peak and patent period compared to untreated animals (Figure 1).

Animals infected with Y strain presented a survival rate of 63% when untreated, and 0% cure rate, Table 1. Treatment with 100 mg/kg/day of BNZ improved the survival rate to 100%, with a cure rate of 75% for the 20-day treatment, which increased to an 87% cure rate when the treatment duration was extended to 40 days. However, for this strain, the lower dose of BNZ (40 mg/kg/day) resulted in no cure for either 20-day or 40-day treatments. Moreover, survival rates with 40 mg/kg/day of BNZ for 40 days decreased with increase in treatment duration (100% survival for 20 days of treatment and 88% for 40 days of treatment).

Untreated animals infected with VL-10 strain presented similar features when the following treatments were compared: BNZ 100 mg/kg/day for 20 days, BNZ 40 mg/kg/day for 20 days, and BNZ 40 mg/kg/day for 40 days (0% cure), Table 1. By changing the time of the standard protocol (BNZ 100 mg/kg/day for 20 days) to BNZ 100 mg/kg/day for 40 days, there is an improvement in therapeutic efficacy (12%). These results indicate that for both strains, Y and VL-10, the therapeutic efficacy is time- and dose-dependent in the acute phase, i.e., the efficacy increases proportionally to the increase in dose or length of treatment.

When infected with the VL-10 strain, all the mice treated in the chronic phase with 100 mg/kg/day for 40 days survived and 62% were cured, Table 2, although when treated with 40 mg/kg/day for 40 days, these animals had already shown cure of 25%, which is a promising result considering the resistance of the evaluated strain. All of the mice infected with the VL-10 strain and untreated survived, but no cure was observed.

**Figure 2:**
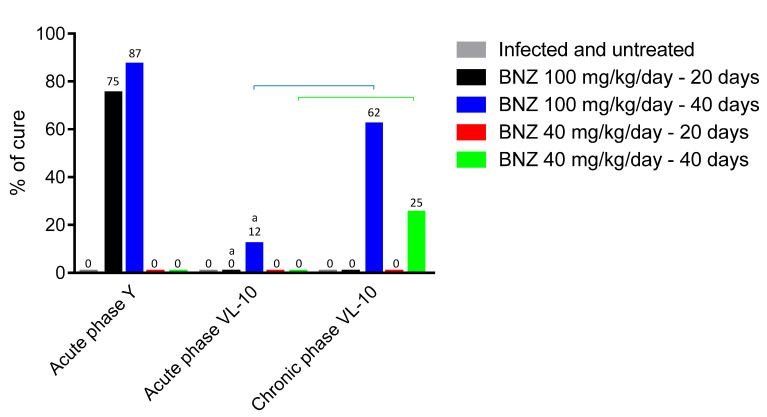
Efficacy of treatment compared between strains and stages of infection. “a” represents significant difference in relation to Acute phase Y. “
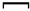
” represents a significant difference among the different phases of infection, but for the same treatment and strain (*p* < 0.05).

When comparing the different strains submitted to the same treatment during the acute phase of infection, the profile of susceptibility to benznidazole found in the literature [23] is maintained: animals infected with the Y strain presented higher percentages of cure, and those infected with the VL-10 strain showed less therapeutic success (Figure 2).

## 4. Discussion

Recently, different works have highlighted the need to review the therapeutic scheme of BNZ, as evidence seems to suggest that the current regime may be an unnecessarily high dose, which too often results in therapy abandonment due to side effects [11,12,13,24]. Thus, we evaluated the efficacy, both on the acute and chronic phases of the disease, of different therapeutic schemes with BNZ, using mice infected with strains that have different BNZ susceptibilities. In addition, since *T. cruzi* infections vary in severity and susceptibility to treatment [23,25], evaluating the effects of different therapeutic protocols in strains with distinct susceptibilities to BNZ could allow more significant results.

In the chronic phase of infection by VL-10 strain, we observed a profile that was dose and time-dependent. The best results were found for animals treated with 100 mg/kg/day for 40 days (standard dose and extended time of treatment); however the treatment with 40 mg/kg/day for 40 days (lower dose with longer time) also obtained good results, although with a lower cure rate than for 100 mg/kg/day for 40 days.

A recent study conducted by Torrico and colleagues evaluated patients with chronic indeterminate Chagas disease [26]. They found that treatment in the chronic phase with a lower dose and shorter treatment duration have similar efficacy when compared to the standard treatment, in addition to higher safety. These results are the contraire found in the present work that showed that the best results are reached with an extend period of treatment. These finding could be related to the profile of resistance of the VL-10 strain to the BZN. Furthermore, pharmacokinetic studies in mice demonstrate that the BNZ plasma concentration remains within the range considered ideal to eliminate intra- and extracellular parasites (3–6 μg/mL) for a short time [27,28], which does not occur in humans [29].

In the acute infection by the Y and VL-10 strains of *T. cruzi* (respectively partly susceptible and resistant)*,* the treatments with a lower dose but standard time of treatment (40 mg/kg/day for 20 days) or a lower dose with longer time (40 mg/kg/day for 40 days) were both unable to promote cure. However, when animals were treated with the standard dose and extended time of treatment (100 mg/kg/day for 40 days) the percentage of cure increased, although without reaching 100% cure in either strain. The need for a higher dose of BNZ for the Y and VL-10 strains is consistent with the fact that they are, respectively, partially resistant and resistant to the drug. In this sense, treatment with BNZ during acute infection in animals infected by Y and VL-10 strains followed the theory of proportionality of BNZ dose and time. These data corroborate with preclinical studies that suggest that treatment in the acute phase of infection by strains partially susceptible or resistant to BNZ is dose- and time- dependent [16]. Mazzeti and collaborators evaluated the standard dose of BNZ (100 mg/kg) with treatment periods varying between 10, 20 or 40 days; and the standard BNZ treatment period (20 days) with doses ranging between 25, 50, 75, 100, and 300 mg/kg/day. However, the study did not evaluate the chronic phase of experimental Chagas disease and the PCR was limited to blood. In addition, we evaluated a lower dose (40 mg/kg/day) during the standard treatment period (20 days) and a longer period (40 days), whereas they evaluated a lower dose (50 mg/kg/day) only using the standard treatment time.

Mice were considered cured only when they presented negative FBE after being subjected to immunosuppression, as well negative blood, colon, and heart qPCR. In the chronic phase of the disease, *T. cruzi* persists in the gastrointestinal tract, mainly in the stomach and colon [30]. When we analyzed fragments of colon of all the mice infected by VL-10 strain, treated or untreated, they were positive in 97% and 25% of the tests in the acute and chronic phases, respectively. Lastly, the acute phase of infection by Y strain showed 53% positivity. Our results demonstrate that independently of the *T. cruzi* strain, the colon is an important place of parasite residence in mice, thus this organ is a crucial place of analysis in post-treatment evaluation. Previous study reports the importance of including colon PCR (combined with blood PCR and FBE after immunosuppression) as one more of the cure criteria for evaluating the effectiveness of compounds in the experimental Chagas disease treatment [31]. Corroborating with our results, the colon presents important tissue parasitism independent of the parasite inoculation pathway (oral or intraperitoneal); in addition, this parasitism is greater in the acute phase when compared to the chronic phase [32].

In relation to qPCR analysis in cardiac tissue of animals infected by VL-10 strain, we found, respectively 84% and 22% positivity in the acute and chronic phases of infection, and for Y strain the results showed 7% positivity in the acute phase of infection. These results were consistent once VL-10 strain is myotropic, and Y strain is macrophagotropic [33,34,35]. In addition, these results are similar to those reported in another study where it was observed better therapeutic report for animals treated with nitroheterocyclics in the chronic phase [36]. This present study concluded, as well as Francisco et al. that, in mice, a chronic *T. cruzi* infection is easier to cure when compared to the acute infection. Francisco et al. using highly sensitive bioluminescence imaging concluded that a possible explanation for this finding would be the fact that the parasite burden in the chronic phase is lower than in the acute phase and restricted to some locations.

Furthermore, using BNZ for a longer period of time can be important for exposing dormant forms to BNZ. In this way, parasites that are actively replicating would be affected at the beginning of the treatment regimen, while the remaining dormant forms would be killed until the end of the treatment [15].

In summary, for experimental Chagas disease: (1) infections by Y strain should be treated with the standard treatment and, where possible, with the standard dose for a longer period (better results); and (2) VL-10 strain infections treated in the acute and chronic phase respond better with the standard dose for a longer time, although a dose reduction with time increased is also an alternative in chronic phase. It is also noted that the treatment of infection by VL-10 strain obtained better results in the chronic phase when compared to the acute phase of the disease.

In relation to methodology the present study used 8 animals per group and this number was determined by sample calculation. Furthermore, in the literature it is common the sample size to range from 6 to 10 animals per group [16,19,37]. In Chagas disease the evaluation of the effectiveness of a treatment is very difficult due to the absence of well-defined cure criteria and in general a combination of different methods is necessary [38,39].

In this study, we opted to use an immunosuppression protocol and subsequent analysis with ESF and blood qPCR [19,20,22]. Similar to other studies, we opted to extend the qPCR assessment to tissues, in order to increase the robustness of the analysis [31,40,41]. The blood PCR is a suitable method to detect therapeutic failure in murine *T. cruzi* infection. However, PCR tests performed on tissues from animals treated with BNZ and considered cured, still detected *T. cruzi* DNA [38]. Therefore, we also perform qPCR in the heart and colon, organs known to be affected by Chagas disease [30,31,42,43].

Taken together, our data indicate that treatment for patients with Chagas disease remains a challenge, as the strains have different results when treated with BNZ in the different phases of infection. Thus, the development of new protocols seems to be a promising route of investigation, and new approaches for therapeutic effectiveness could include strain characterization, potentiating discovery of biomarkers of resistance that will allow individualized treatment, as well as the discovery of new drugs.

## Figures and Tables

**Table 1:** Time- and dose-dependence of BNZ treatment on different *Trypanosoma cruzi* strains in the acute phase of Chagas disease.

Strain ^£^	Group	Survival ^§^	Positive	Positive qPCR ^ɸ^			Total Positive	Cure ^#^
			FBE ^¶^	Blood	Heart	Colon	Mice	
Y	Untreated	5/8 (63%)	5/5 (100%)	*	-	-	5/5 (100%)	0
	BNZ 100 mg/kg/day—20 days	8/8 (100%)	0/8 (0%)	1/8 (13%)	1/8 (13%)	2/8 (25%)	2/8 (25%)	75 ^a,d,e^
	BNZ 100 mg/kg/day—40 days	8/8 (100%)	1/8 (22%)	0/7 (0%)	0/8 (0%)	0/8 (0%)	1/8 (13%)	87 ^a,b,d,e^
	BNZ 40 mg/kg/day—20 days	8/8 (100%)	7/8 (88%)	1/1 (100%)	1/7 (14%)	7/7 (100%)	8/8 (100%)	0
	BNZ 40 mg/kg/day—40 days	7/8 (88%)	7/7 (100%)	-	0/7 (0%)	7/7 (100%)	7/7 (100%)	0 ^b^
								
VL-10	Untreated	8/8 (100%)	8/8 (100%)	-	8/8 (100%)	8/8 (100%)	8/8 (100%)	0
	BNZ 100 mg/kg/day—20 days	7/8 (88%)	6/7 (88%)	0/1 (0%)	5/7 (71%)	7/7 (100%)	7/7 (100%)	0
	BNZ 100 mg/kg/day—40 days	8/8 (100%)	5/8 (63%)	0/3 (0%)	4/8 (50%)	7/8 (88%)	7/8 (88%)	12 ^a,b,d,e^
	BNZ 40 mg/kg/day—20 days	8/8 (100%)	8/8 (100%)	-	8/8 (100%)	8/8 (100%)	8/8 (100%)	0
	BNZ 40 mg/kg/day—40 days	7/8 (88%)	7/7 (100%)	-	6/6 ** (100%)	6/6 (100%)	7/7 (100%)	0

(*) All mice died with positive FBE; (**) 1 mouse died after presenting positive FBE. ^£^ Swiss female mice were inoculated with 5 × 10^3^ trypomastigotes of the Y, and VL-10 strains. ^§^ Up to 30 days after treatment. ^¶^ Fresh blood exam (FBE) after cyclophosphamide immunosuppression. ^ɸ^ Quantitative real-time polymerase chain reaction (qPCR) after cyclophosphamide immunosuppression. Blood qPCR was performed 5 days after immunosuppression, only for negative FBE assays. Tissue qPCR was performed 30 days after immunosuppression for all mice. ^#^ Cured: when all tests (FBE and qPCR) were negative. Letters represent a significant difference in cure rates when compared to: “a” Untreated group; “b” BNZ 100 mg/kg/day—20 days; “d” BNZ 40 mg/kg/day—20 days and “e” BNZ 40 mg/kg/day—40 days.

**Table 2:** Time- and dose-dependence of BNZ treatment on VL-10 *Trypanosoma cruzi* strain in the chronic phase of Chagas disease.

Strain ^£^	Group	Survival ^§^	Positive	Positive qPCR ^ɸ^			Total	Cure ^#^
			FBE ^¶^	Blood	Heart	Colon	Positive mice	(%)
VL-10	Untreated	8/8 (100%)	5/8 (63%)	2/3 (67%)	4/5 * (80%)	2/5 (40%)	8/8 (100%)	0
	BNZ 100 mg/kg/day—20 days	8/8 (100%)	5/8 (63%)	2/3 (67%)	2/7 * (29%)	4/7 (57%)	8/8 (100%)	0
	BNZ 100 mg/kg/day—40 days	8/8 (100%)	2/8 (25%)	0/6 (0%)	0/8 (0%)	1/8 (13%)	3/8 (38%)	62 ^a,b^
	BNZ 40 mg/kg/day—20 days	8/8 (100%)	4/8 (50%)	3/4 (75%)	1/7 * (14%)	1/7 (14%)	8/8 (100%)	0 ^c^
	BNZ 40 mg/kg/day—40 days	8/8 (100%)	6/8 (75%)	0/2 (0%)	0/5 * (0%)	0/5 (0%)	6/8 (75%)	25 ^a,b,c,d^

(*) Mice died with positive FBE. ^£^ Swiss female mice were inoculated with 5 × 10^3^ trypomastigotes of the VL-10 strains and were treated 120 days after infection. ^§^ Up to 30 days after treatment. ^¶^ Fresh blood exam (FBE) after cyclophosphamide immunosuppression. ^ɸ^ Quantitative real-time polymerase chain reaction (qPCR) after cyclophosphamide immunosuppression. Blood qPCR was performed 5 days after immunosuppression, only for negative FBE assays. Tissue qPCR was performed 30 days after immunosuppression for all mice. ^#^ Cured: when all tests (FBE and qPCR) were negative. Letters represent a significant difference in cure rates when compared to: “a” Untreated group; “b” BNZ 100 mg/kg/day—20 days; “c” BNZ 100 mg/kg/day—40 days; “d” BNZ 40 mg/kg/day—20 days.

## Data Availability

Not applicable.
